# Supplementary material for: Evaluating Sequencing Strategies for Endometrial Microbiome Profiling in Endometrial Cancer: A Comparative Study of Short‐ and Long‐Read 16S rRNA Approaches
Source: Cancer Rep (Hoboken). 2026 Apr 14;9(4):e70540. doi: 10.1002/cnr2.70540 (PMC13079076; doi:10.1002/cnr2.70540)
Supplement: Supplementary file 2 — Figure S2: Alpha rarefaction analysis of Illumina and ONT sequencing data. Alpha rarefaction plots showing genus‐level microbial diversity for (a) Illumina and (b) Oxford Nanopore Technologies datasets. The dotted line indicates the read depth used for diversity analysis: 10 000 reads for Illumina and 140 000 reads for ONT. [file CNR2-9-e70540-s003.docx]

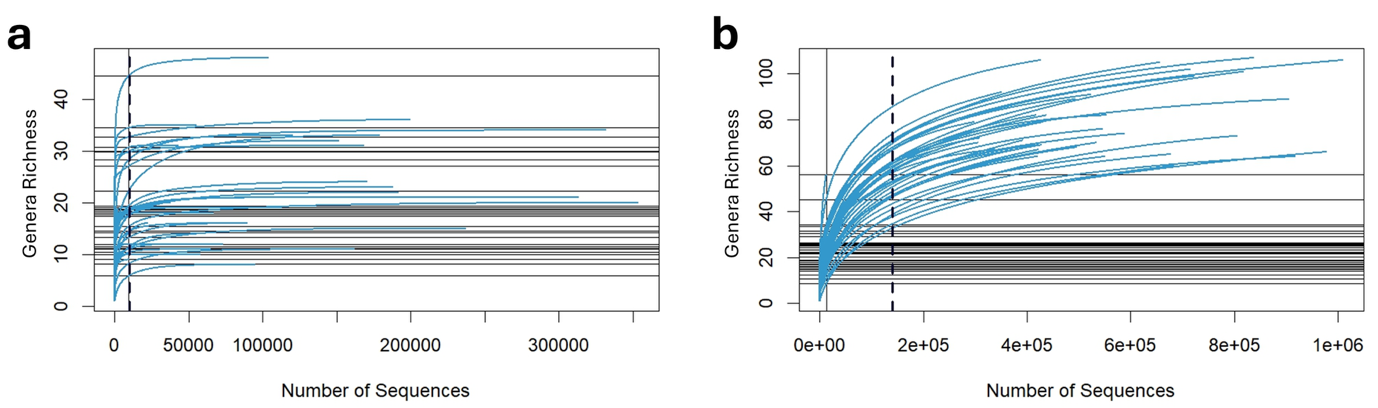


**Figure S2.** Alpha rarefaction analysis of Illumina and ONT sequencing data. Alpha rarefaction plots showing genus-level microbial diversity for (a) Illumina and (b) Oxford Nanopore Technologies datasets. The dotted line indicates the read depth used for diversity analysis: 10,000 reads for Illumina and 140,000 reads for ONT.
