# Supplementary material for: Evaluating Sequencing Strategies for Endometrial Microbiome Profiling in Endometrial Cancer: A Comparative Study of Short‐ and Long‐Read 16S rRNA Approaches
Source: Cancer Rep (Hoboken). 2026 Apr 14;9(4):e70540. doi: 10.1002/cnr2.70540 (PMC13079076; doi:10.1002/cnr2.70540)
Supplement: Supplementary file 4 — Figure S4: Comparison of microbial diversity by sample site. (a) Violin plots showing alpha diversity metrics—Observed, Shannon, and Simpson indices, based on genus‐level data, comparing samples from the uterus (blue, n = 38) and vagina (red, n = 3). Each dot represents an individual sample. Samples were rarefied to 140 000 reads, and unassigned reads were excluded. Statistical significance was assessed using the Wilcoxon test: p < 0.05 (*), p < 0.01 (**). (b) Principal Coordinate Analysis (PCoA) plots based on Bray–Curtis dissimilarity, comparing microbial communities between uterus (blue, n = 38) and vagina (red, n = 3) swabs at the genus level. Each dot represents an individual sample, labelled by the patient letter. The ellipse indicates the 95% confidence interval for uterus samples. Samples were rarefied as above, and unassigned reads were excluded. PERMANOVA test results: F = 3.08, R 2 = 0.07491, p = 0.003 (999 permutations). [file CNR2-9-e70540-s001.docx]

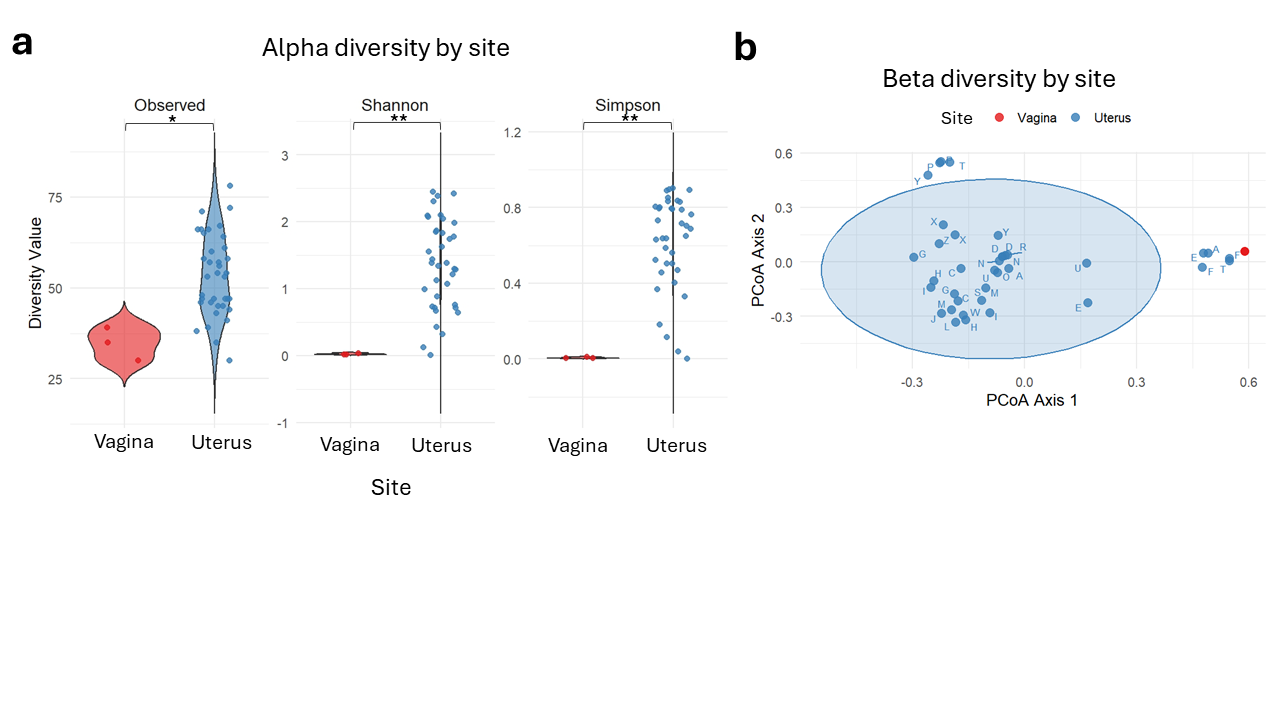


Figure S4. Comparison of microbial diversity by sample site. (a) Violin plots showing alpha diversity metrics - Observed, Shannon, and Simpson indices, based on genus-level data, comparing samples from the uterus (blue, n = 38) and vagina (red, n = 3). Each dot represents an individual sample. Samples were rarefied to 140,000 reads, and unassigned reads were excluded. Statistical significance was assessed using the Wilcoxon test: p < 0.05 (*), p < 0.01 (**). (b) Principal Coordinate Analysis (PCoA) plots based on Bray-Curtis dissimilarity, comparing microbial communities between uterus (blue, n = 38) and vagina (red, n = 3) swabs at the genus level. Each dot represents an individual sample, labelled by the patient letter. The ellipse indicates the 95% confidence interval for uterus samples. Samples were rarefied as above, and unassigned reads were excluded. PERMANOVA test results: F = 3.08, R^2^ = 0.07491, p = 0.003 (999 permutations).
