# Supplementary material for: Evaluating Sequencing Strategies for Endometrial Microbiome Profiling in Endometrial Cancer: A Comparative Study of Short‐ and Long‐Read 16S rRNA Approaches
Source: Cancer Rep (Hoboken). 2026 Apr 14;9(4):e70540. doi: 10.1002/cnr2.70540 (PMC13079076; doi:10.1002/cnr2.70540)
Supplement: Supplementary file 5 — Figure S5: Principal component analysis of ONT samples by sample site. Principal Component Analysis (PCA) plot based on Hellinger‐transformed genus‐level data from ONT sequencing. Samples were derived from either the uterus (blue, n = 38) or vagina (red, n = 3). Uterus samples are labelled by the patient letter. [file CNR2-9-e70540-s005.docx]

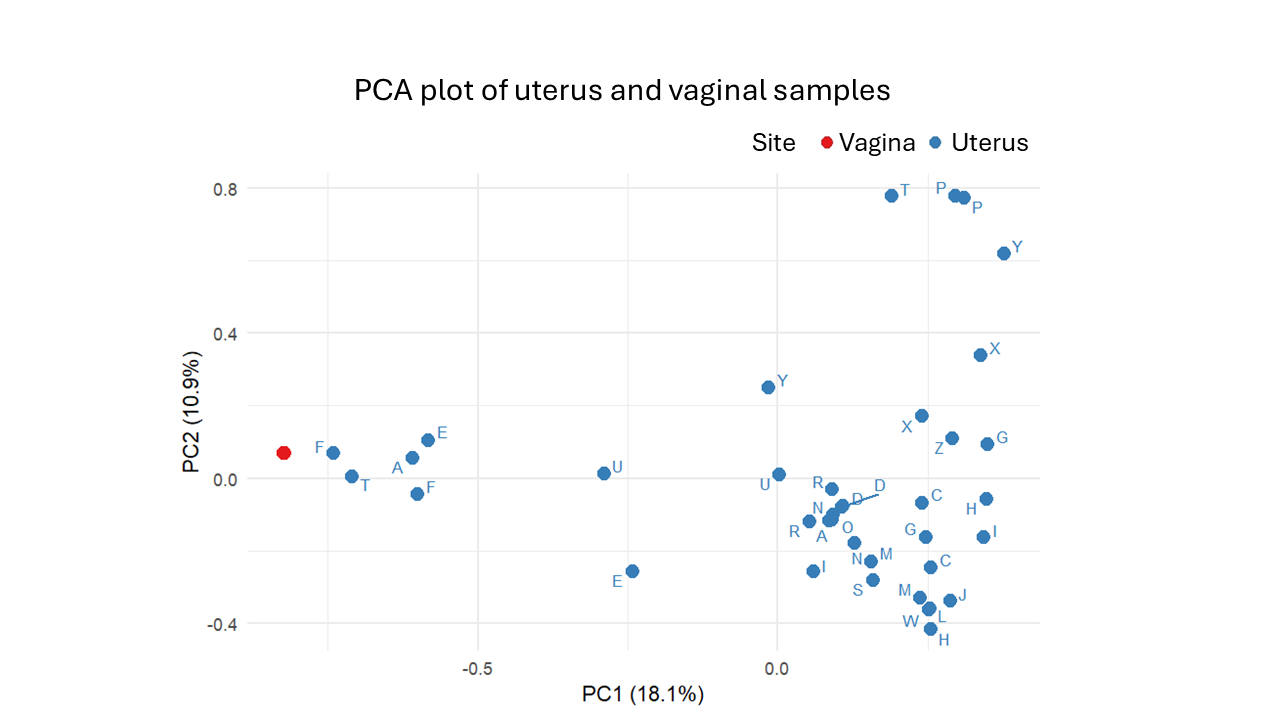


**Figure S5.** Principal component analysis of ONT samples by sample site. Principal Component Analysis (PCA) plot based on Hellinger-transformed genus-level data from ONT sequencing. Samples were derived from either the uterus (blue, *n* = 38) or vagina (red, *n* = 3). Uterus samples are labelled by the patient letter.
