# Supplementary material for: Evaluating Sequencing Strategies for Endometrial Microbiome Profiling in Endometrial Cancer: A Comparative Study of Short‐ and Long‐Read 16S rRNA Approaches
Source: Cancer Rep (Hoboken). 2026 Apr 14;9(4):e70540. doi: 10.1002/cnr2.70540 (PMC13079076; doi:10.1002/cnr2.70540)
Supplement: Supplementary file 8 — Figure S8: Comparison of alpha and beta diversity between Illumina and ONT sequencing platforms at the genus level. (a) Alpha Diversity Comparison: Violin plots of Observed, Shannon, and Simpson indices at the genus level, comparing Illumina (light blue) and ONT (dark blue) sequencing platforms. Each dot represents an individual sample. Samples were rarefied to 10 000 reads, excluding unassigned reads. Statistical significance was assessed using paired Wilcoxon rank‐sum tests (*p < 0.05, **p < 0.01, adjusted for multiple comparisons). (b) Beta Diversity (Bray‐Curtis PCoA): Principal coordinate analysis of Bray–Curtis dissimilarity at the genus level, comparing Illumina and ONT platforms. Each dot represents a sample, labelled by the patient letter. Dotted lines connect matched samples sequenced on both platforms. Ellipses indicate 95% confidence intervals. Samples were rarefied as above, excluding unassigned reads. PERMANOVA yielded F = 10.057, R 2 = 0.123, p = 0.001 (999 permutations). [file CNR2-9-e70540-s010.docx]

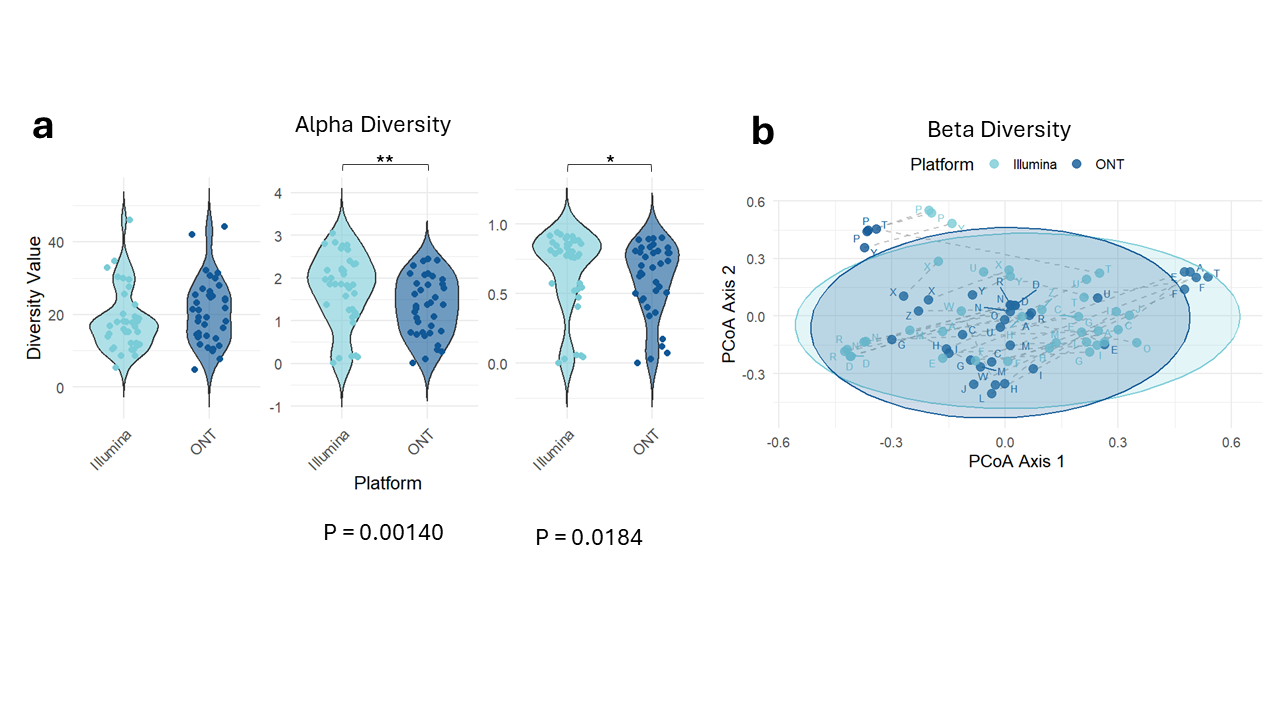


**Figure S8.** Comparison of alpha and beta diversity between Illumina and ONT sequencing platforms at the genus level. (a) Alpha Diversity Comparison: Violin plots of Observed, Shannon, and Simpson indices at the genus level, comparing Illumina (light blue) and ONT (dark blue) sequencing platforms. Each dot represents an individual sample. Samples were rarefied to 10,000 reads, excluding unassigned reads. Statistical significance was assessed using paired Wilcoxon rank-sum tests (*p < 0.05, **p < 0.01, adjusted for multiple comparisons). (b) Beta Diversity (Bray-Curtis PCoA): Principal coordinate analysis of Bray-Curtis dissimilarity at the genus level, comparing Illumina and ONT platforms. Each dot represents a sample, labelled by the patient letter. Dotted lines connect matched samples sequenced on both platforms. Ellipses indicate 95% confidence intervals. Samples were rarefied as above, excluding unassigned reads. PERMANOVA yielded F = 10.057, R^2^ = 0.123, *p* = 0.001 (999 permutations).
