# Supplementary material for: Evaluating Sequencing Strategies for Endometrial Microbiome Profiling in Endometrial Cancer: A Comparative Study of Short‐ and Long‐Read 16S rRNA Approaches
Source: Cancer Rep (Hoboken). 2026 Apr 14;9(4):e70540. doi: 10.1002/cnr2.70540 (PMC13079076; doi:10.1002/cnr2.70540)
Supplement: Supplementary file 9 — Figure S9: Hellinger‐transformed PCA of uterus samples and negative controls. Principal Component Analysis (PCA) plot based on Hellinger‐transformed genus‐level data from ONT sequencing. The plot compares uterus samples collected for platform comparison (blue, n = 38) with two types of negative controls: no template control (green) added during the sequencing process and processing control (purple) added prior to DNA isolation and quantification. Uterus samples are labelled by patient letter. [file CNR2-9-e70540-s008.docx]

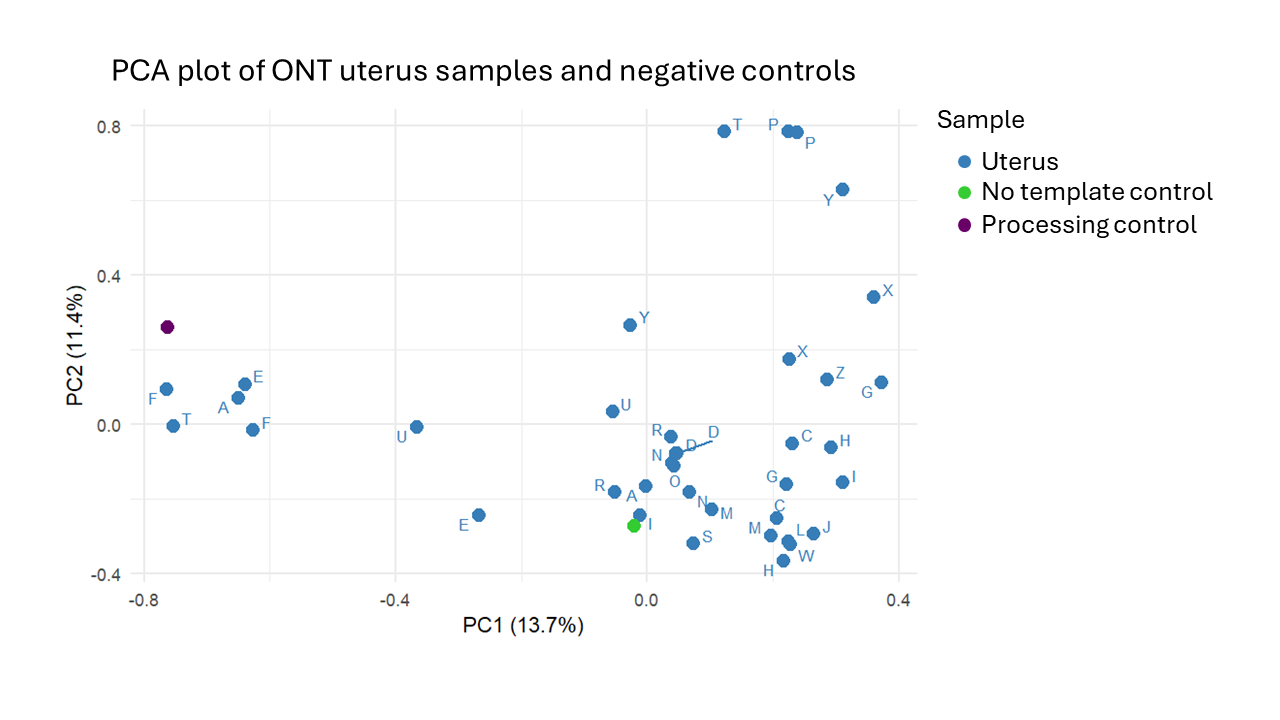


**Figure S9.** Hellinger-transformed PCA of uterus samples and negative controls. Principal Component Analysis (PCA) plot based on Hellinger-transformed genus-level data from ONT sequencing. The plot compares uterus samples collected for platform comparison (blue, *n* = 38) with two types of negative controls: no template control (green) added during the sequencing process and processing control (purple) added prior to DNA isolation and quantification. Uterus samples are labelled by patient letter.
