# Supplementary material for: Evaluating Sequencing Strategies for Endometrial Microbiome Profiling in Endometrial Cancer: A Comparative Study of Short‐ and Long‐Read 16S rRNA Approaches
Source: Cancer Rep (Hoboken). 2026 Apr 14;9(4):e70540. doi: 10.1002/cnr2.70540 (PMC13079076; doi:10.1002/cnr2.70540)
Supplement: Supplementary file 10 — Figure S10: Comparisons of endometrial swab DNA output between different collection and storage methods (A–D). (a) The ratio of bacteria to human DNA copies using ddPCR. (b) The total DNA concentration in each sample. [file CNR2-9-e70540-s006.docx]

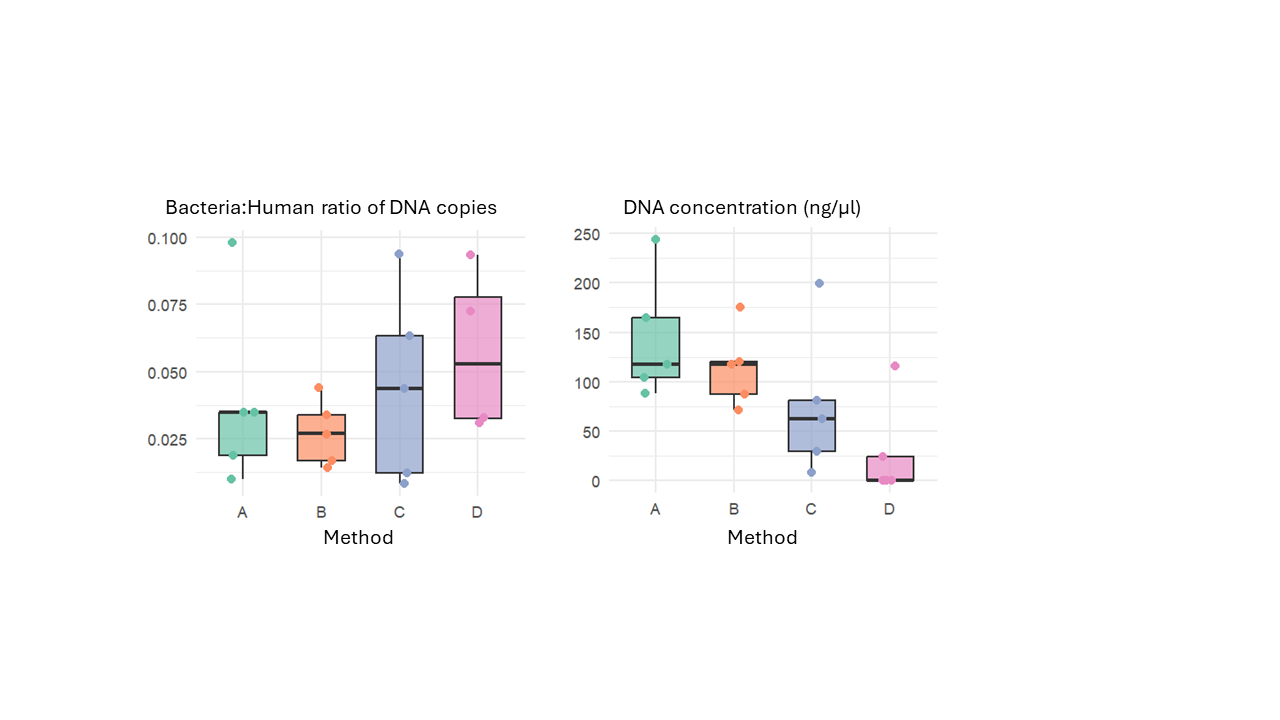


**Figure S10.** Comparisons of endometrial swab DNA output between different collection and storage methods (A-D). (a) The ratio of bacteria to human DNA copies using ddPCR. (b) The total DNA concentration in each sample.
