# Supplementary material for: Evaluating Sequencing Strategies for Endometrial Microbiome Profiling in Endometrial Cancer: A Comparative Study of Short‐ and Long‐Read 16S rRNA Approaches
Source: Cancer Rep (Hoboken). 2026 Apr 14;9(4):e70540. doi: 10.1002/cnr2.70540 (PMC13079076; doi:10.1002/cnr2.70540)
Supplement: Supplementary file 11 — Figure S11: Summary of the average number of reads assigned to genus (from ONT data) and copies of bacterial DNA isolated using processing and storage methods A–D. [file CNR2-9-e70540-s002.docx]

| **Method** | **Reads assigned at genus level (%)** | **Mean bacterial DNA copies per 5 ng sample** | **Standard deviation of bacterial DNA copies per 5 ng sample** |
| --- | --- | --- | --- |
| A | 80.42 | 11.8 | 2.64 |
| B | 75.07 | 11.6 | 4.96 |
| C | 78.07 | 12.2 | 7.11 |
| D | 78.07 | 24.8 | 10.64 |

**Figure S11.** Summary of the average number of reads assigned to genus (from ONT data) and copies of bacterial DNA isolated using processing and storage methods A-D.
