# Supplementary material for: Evaluating Sequencing Strategies for Endometrial Microbiome Profiling in Endometrial Cancer: A Comparative Study of Short‐ and Long‐Read 16S rRNA Approaches
Source: Cancer Rep (Hoboken). 2026 Apr 14;9(4):e70540. doi: 10.1002/cnr2.70540 (PMC13079076; doi:10.1002/cnr2.70540)
Supplement: Supplementary file 12 — Figure S12: Alpha rarefaction analysis of ONT samples by collection and storage method. Alpha rarefaction plots showing genus‐level microbial diversity in ONT‐sequenced samples used to compare different collection and storage methods. The dotted line at 130 000 reads indicates the read depth used for diversity analysis. [file CNR2-9-e70540-s009.docx]

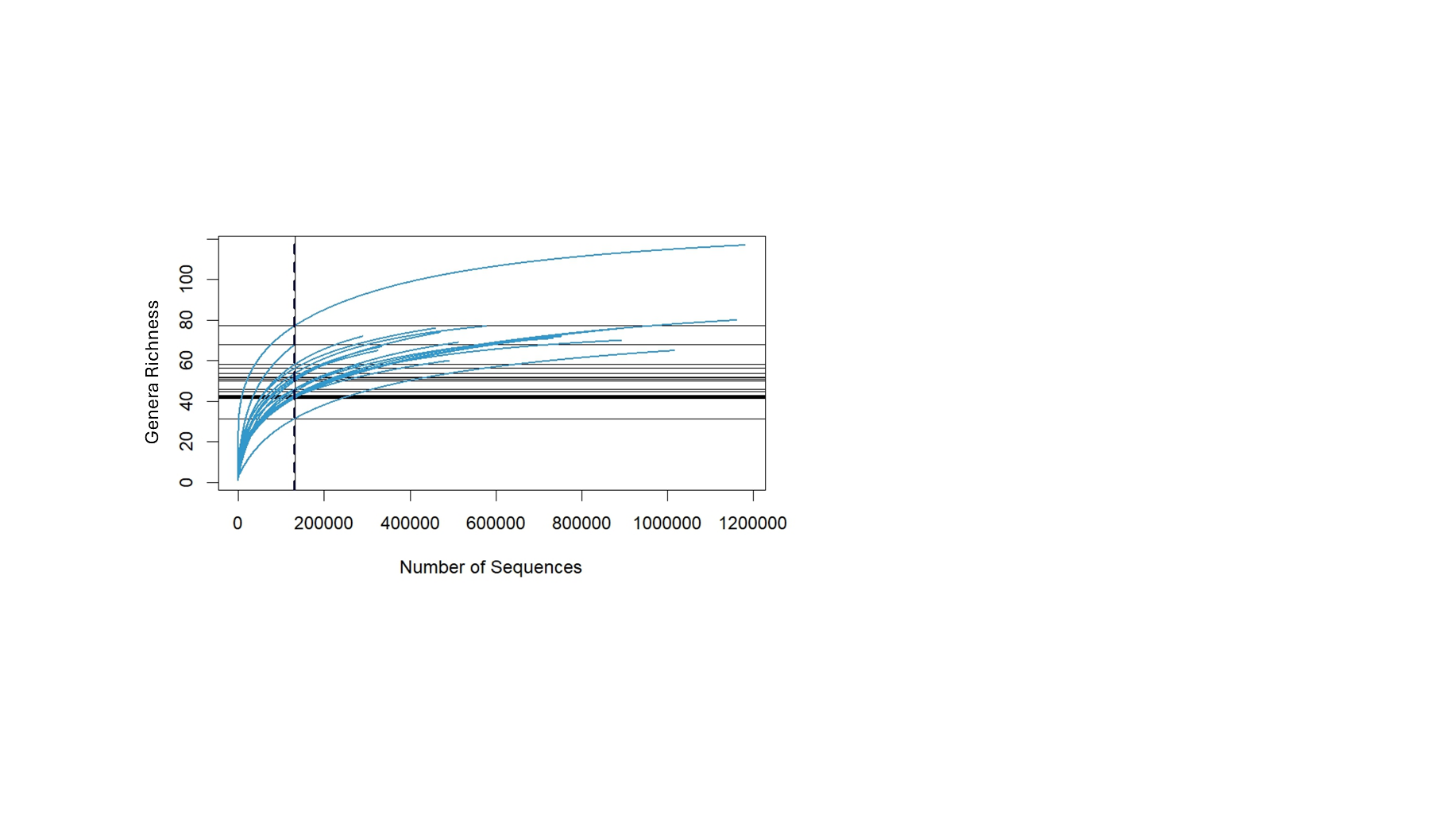


**Figure S12.** Alpha rarefaction analysis of ONT samples by collection and storage method. Alpha rarefaction plots showing genus-level microbial diversity in ONT-sequenced samples used to compare different collection and storage methods. The dotted line at 130,000 reads indicates the read depth used for diversity analysis.
