# Supplementary material for: Evaluating Sequencing Strategies for Endometrial Microbiome Profiling in Endometrial Cancer: A Comparative Study of Short‐ and Long‐Read 16S rRNA Approaches
Source: Cancer Rep (Hoboken). 2026 Apr 14;9(4):e70540. doi: 10.1002/cnr2.70540 (PMC13079076; doi:10.1002/cnr2.70540)
Supplement: Supplementary file 13 — Figure S13: Microbial diversity across patients using ONT sequencing. (a) Box plots showing alpha diversity metrics—Observed, Shannon, and Simpson indices, based on genus‐level data for samples collected from patients 1–5. Each dot represents an individual sample. Samples were rarefied to 130 000 reads, and unassigned reads were excluded. Statistical significance was assessed using the Kruskal–Wallis test, followed by Dunn's post hoc test. p < 0.05 (*) adjusted for multiple tests. (b) Principal Coordinate Analysis (PCoA) plots based on Bray–Curtis dissimilarity, comparing microbial community composition across patients 1–5 at the genus level. Each dot represents an individual sample, labelled by the method used for sample processing and storage (A–D). Ellipses represent the 95% confidence interval for each patient group, excluding patient 2, which had too few samples for ellipse calculation. Samples were rarefied as above, and unassigned reads were excluded. Pairwise PERMANOVA tests revealed significant differences between: Patients 1 and 5 (R 2 = 0.398, p = 0.029), Patients 2 and 5 (R 2 = 0.410, p = 0.035), Patients 3 and 5 (R 2 = 0.430, p = 0.029), Patients 4 and 5 (R 2 = 0.310, p = 0.028). [file CNR2-9-e70540-s014.docx]

**
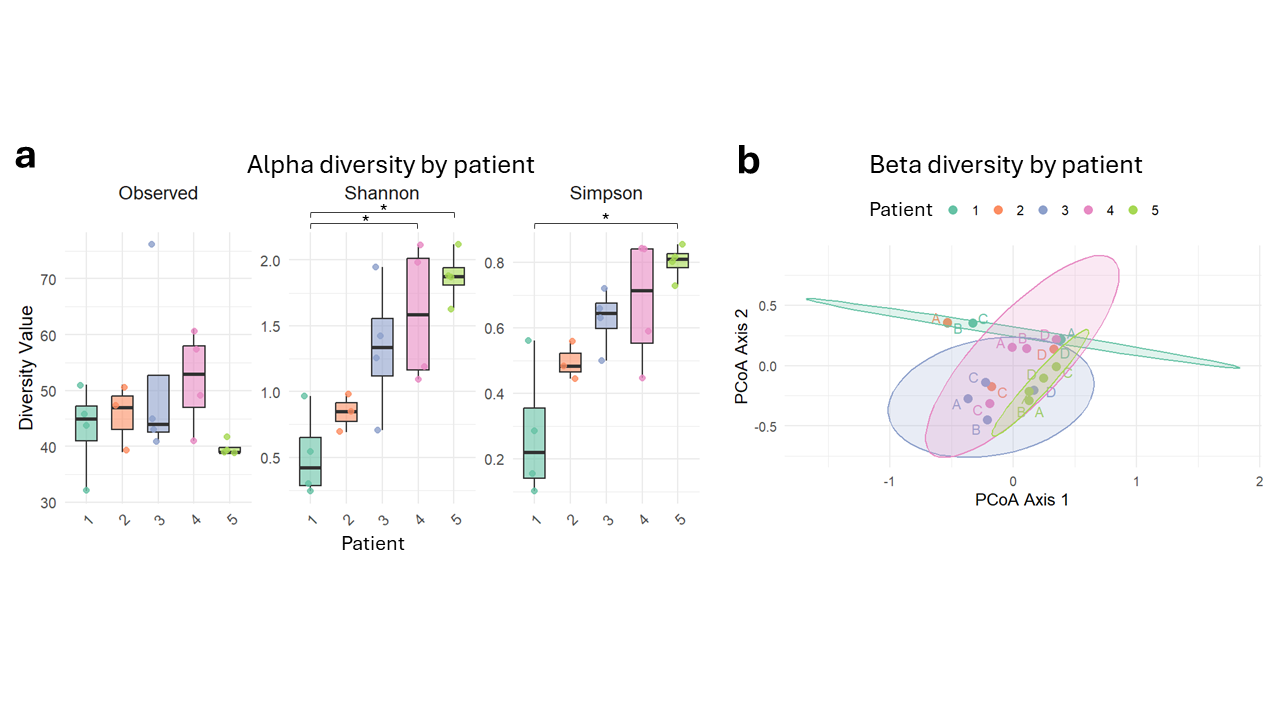
**

**Figure S13.** Microbial diversity across patients using ONT sequencing. (a) Box plots showing alpha diversity metrics - Observed, Shannon, and Simpson indices, based on genus-level data for samples collected from patients 1-5. Each dot represents an individual sample. Samples were rarefied to 130,000 reads, and unassigned reads were excluded. Statistical significance was assessed using the Kruskal-Wallis test, followed by Dunn’s post hoc test. *p* < 0.05 (*) adjusted for multiple tests. (b) Principal Coordinate Analysis (PCoA) plots based on Bray-Curtis dissimilarity, comparing microbial community composition across patients 1-5 at the genus level. Each dot represents an individual sample, labelled by the method used for sample processing and storage (A-D). Ellipses represent the 95% confidence interval for each patient group, excluding patient 2, which had too few samples for ellipse calculation. Samples were rarefied as above, and unassigned reads were excluded. Pairwise PERMANOVA tests revealed significant differences between: Patients 1 and 5 (R^2^ = 0.398, *p* = 0.029), Patients 2 and 5 (R^2^ = 0.410, *p* = 0.035), Patients 3 and 5 (R^2^ = 0.430, *p* = 0.029), Patients 4 and 5 (R^2^ = 0.310, *p* = 0.028).
